# Cortico-cortical communication dynamics

**DOI:** 10.3389/fnsys.2014.00019

**Published:** 2014-05-05

**Authors:** Per E. Roland, Claus C. Hilgetag, Gustavo Deco

**Affiliations:** ^1^Department of Neuroscience and Pharmacology, Faculty of Health Sciences, University of CopenhagenCopenhagen, Denmark; ^2^Department of Computational Neuroscience, University Medical Center Hamburg-EppendorfHamburg, Germany; ^3^Department of Health Sciences, Boston UniversityBoston, MA, USA; ^4^Department of Technology, University of Pompeu FabraBarcelona, Spain

**Keywords:** spontaneous activity, synaptic transmission, membrane potential dynamics, spiking dynamics, cortical areas

## Abstract

In principle, cortico-cortical communication dynamics is simple: neurons in one cortical area communicate by sending action potentials that release glutamate and excite their target neurons in other cortical areas. In practice, knowledge about cortico-cortical communication dynamics is minute. One reason is that no current technique can capture the fast spatio-temporal cortico-cortical evolution of action potential transmission and membrane conductances with sufficient spatial resolution. A combination of optogenetics and monosynaptic tracing with virus can reveal the spatio-temporal cortico-cortical dynamics of specific neurons and their targets, but does not reveal how the dynamics evolves under natural conditions. Spontaneous ongoing action potentials also spread across cortical areas and are difficult to separate from structured evoked and intrinsic brain activity such as thinking. At a certain state of evolution, the dynamics may engage larger populations of neurons to drive the brain to decisions, percepts and behaviors. For example, successfully evolving dynamics to sensory transients can appear at the mesoscopic scale revealing how the transient is perceived. As a consequence of these methodological and conceptual difficulties, studies in this field comprise a wide range of computational models, large-scale measurements (e.g., by MEG, EEG), and a combination of invasive measurements in animal experiments. Further obstacles and challenges of studying cortico-cortical communication dynamics are outlined in this critical review.

## Background and scope

When one speaks of cortico-cortical connections, one usually means that axons start in one cortical area and end in another cortical area. These cortico-cortical axons are excitatory, releasing glutamate at their terminals (Ottersen and Storm-Mathisen, 1986). Neurons communicate by sending an action potential or a sequence of action potentials, *r*(*t*), down their axons. By *cortico-cortical communication*, we mean that the *r*(*t*) travels from one neuron in one cortical area, area A, to target neurons in another cortical area, area B. Area A often has different sets of output neurons, such that set 1 send the *r*(*t*)s produced to area B, set 2 to area C and so on. In this way the *r*(*t*)s produced by the neurons in area A are communicated to target neurons in several other areas (Felleman and van Essen, 1991; Scannell and Young, 1993). Each area has a unique pattern of connections (Passingham et al., 2002). The word communicate does not imply that the neurons in one area send coded messages to their target neurons; it simply means that the neurons send action potentials to the pre-synaptic terminals on the target neurons. As cortico-cortical neurons are excitatory, the glutamate release increases the currents flowing through the membranes of the target neurons, d*V*_m_(*t*)/d*t*, such that this term becomes net-positive, no matter whether the target neurons are excitatory or inhibitory. Thus

(1) $$r{\left(t\right)}_{area}{}_{A}\Rightarrow dV_{m}{\left(t\right)}_{area}{}_{B}/dt↑$$

in which *V*_m_(*t*) is the membrane potential. Note that for each pre-synaptic site, the cortico-cortical communication is transmission over one synaptic cleft only. The increased excitation of the target neurons may or may not lead to action potentials in area B. The point is that the communication of excitation to target neurons in area B, as a minimum, changes the membrane dynamics of the target neurons in area B, which may influence the further spiking in area B.

As neurons in one area communicate *r*(*t*)s to several areas, one could imagine that d*V*_m_(*t*)/d*t* would increase in several cortical areas when the *r*(*t*)s are transmitted. Moreover, as some neurons in the target areas may fire *r*(*t*)s as a consequence of the communication, these neurons might excite other neurons within the target area, of which some might communicate to another set of target areas. This should evoke d*V*_m_(*t*)/d*t* increases in yet other areas. By *cortico-cortical communication dynamics* we mean the spatial and temporal evolution of *r*(*t*)s and d*V*_m_(*t*)/d*t* between neurons in different cortical areas. If we could measure how such cortico-cortical communications evolve, then we may understand the mechanisms that ultimately drive the cerebral cortex and the brain to particular percepts and behaviors. Thus we would have captured essential traits of how the brain works in a relevant time scale and relevant spatial scale.

Despite the theoretical simplicity, experimental studies of cortico-cortical communication dynamics meet many and complicated obstacles. First, as the relevant time scale is 0.5 ms or less, many methods based on slower brain signals are automatically excluded, for example blood oxygen level detection (BOLD) responses, intrinsic optical signals, regional cerebral blood flow and metabolism and other methods based on vascular kinetics and extracellular diffusion over larger distances positron emission tomography (PET). Second, the relevant spatial scale ranges from single dendrites to the whole cortex. Current methods with sufficient time resolution are in practical use limited to certain spatial scales. At a small spatial scale, voltage sensitive dye recordings can capture events at the single dendrite and single neuron scale (Canepari et al., 2010; Fisher and Salzberg, 2010). At a large spatial scale, magnetoencephalography (MEG) captures events over the whole cortex, albeit with some limitations. It is a major theoretical and practical challenge to combine these methods. Furthermore, *in vivo*, both methods are largely insensitive to action potentials (Hämäläinen et al., 1993; Petersen et al., 2003a; Grinvald and Hildseheim, 2004). Action potentials can be captured easily with electrodes near the axon hillock; but so far there is no method by which on can capture all action potentials in the brain. Ca^2+^ sensitive dyes can be used to localize neurons that *had* communicated action potentials, but current dyes are too slow to capture the time when each potential is created (Grienberger and Konnerth, 2012). In a prominent recent proposal, neuroscientists describe new (nano-) technologies that may allow capturing every single action potential in the cortex of the mouse within the next 15 years (Alivisatos et al., 2012).

A test of cortico-cortical transmission of *r*(*t*) from one cortical area to another requires two electrodes. One electrode, very close to or into the transmitting neuron, recording the action potentials transmitted and one electrode into one of the target neurons in the receiving area to record the increase in d*V*_m_(*t*)/d*t* and eventual subsequent action potentials. This monosynaptic transmission then in most cases should take a few ms until the d*V*_m_(*t*)/d*t* increases. One problem with this strategy is that the transmitting axon most likely makes synapses on the dendrites of the target neurons. Depending on where on the dendrites the transmitter opens the ion channels, it may take up to 5–6 ms until the dendritic d*V*_m_(*t*)/d*t* increase is detected at the soma where the electrode is sampling. This is because the dendritic conduction velocities are around 0.1 mm ms^−1^ (Nicoll et al., 1993; Stuart and Spruston, 1998). Actually there might not even be a detectable d*V*_m_(*t*)/d*t* increase, as this could be shunted out by prevailing or concomitant inhibitory conductances and conductances provided by the many other (in the order of 1000 or more) neurons that make synapses on the target neuron. Now, the chance of putting a patch electrode into precisely one of the dendrites that receive the glutamate from the transmitting neuron is very small indeed. One may object that sub-threshold excitation of dendrites does not matter anyway, only if the target neurons spike they can change the dynamics. This does not seem to be the case, as sub-threshold d*V*_m_(*t*)/d*t* increases very well may influence the subsequent dynamics of a neuron population both in single cortical neurons and at the mesoscopic neuron network scale. Indeed such d*V*_m_(*t*)/d*t* increases can be induced by neurons in other cortical areas (Roland et al., 2006; Ahmed et al., 2008; Harvey et al., 2009; Niell and Stryker, 2010; Roland, 2010; Harvey and Roland, 2013; Zagha et al., 2013).

Electrical stimulation and later, cortical micro-stimulation has been used widely to examine cortico-cortical communications. However, unless the micro-stimulation is done intracellularly, a small population of neurons is usually excited. Furthermore, even moderate stimulation currents evoke inhibition in the target area, most likely from engaging inhibitory neurons in the target area (Kara et al., 2002; Logothetis et al., 2010). It is possible to detect monosynaptic transmission between two areas by antidromic electrical stimulation of axons, for example those axons running from the primary visual area 17 to area middle temporal lobe visual area (MT)/V5, for which the time of transmission is 2 ms (Movshon and Newsome, 1996). This is an elegant technique, in which the synaptic transmission is checked by colliding the antidromic action potential with a sensory evoked orthodromic action potential, giving undoubtedly valuable results. However, even this method does not give any further information on the evolving dynamics associated with *natural* use of this communication. Similarly, although there now are powerful methods to localize the group of neurons that connect monosynaptically to a neuron of interest (Wickersham et al., 2007; Wall et al., 2010), the mere proof of the monosynaptic connection cannot predict how the d*V*_m_(*t*)/d*t* and inter-area spike dynamics will evolve under natural circumstances. Furthermore, although it is possible to stimulate neurons electrically by micro-stimulation, and although it is possible to stimulate genetically modified neurons by laser beam pulses, it is the naturally evolving *r*(*t*) and membrane potential spatio-temporal dynamics that is in the focus when scientists want to understand how the cerebral cortex creates perception and behavior (Lim et al., 2012). Identification of target neurons, measurements of conduction velocities and other reductionist approaches still might be very helpful in constraining the interpretation of cortico-cortical dynamics under natural conditions.

The study of cortico-cortical communication dynamics would be so much easier if only a certain spatial scale mattered. As one could imagine, the dynamics must at a certain state of its evolution engage larger populations of cortical neurons, as only larger populations may drive the brain to a certain percept or towards a certain behavior. Consequently, all dynamics of the *r*(*t*) and d*V*_m_(*t*)/d*t* that matters may occur at the (mesoscopic) scale of neuron populations. Unfortunately, the *r*(*t*) of a single neuron may change also the *r*(*t*) and d*V*_m_(*t*)/d*t* dynamics of larger neuron populations. Consequently, it seems that one must keep track of every neuron to understand the evolution of cortico-cortical communication dynamics. This seems so in both experiments and reasonable realistic models of the brain (Houweling and Brecht, 2008; Izhikevich and Edelman, 2008; London et al., 2010).

## Spontaneous and intrinsic communication dynamics—experiments and computational modeling

**Figure 1:**
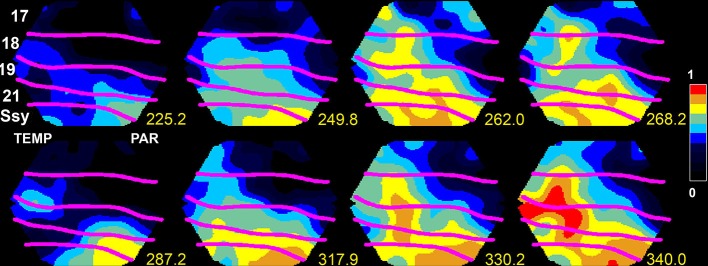
**Upstate in areas SSy and 21 spreading to lower visual areas 18 and 17 in the ferret**. The voltage sensitive dye signal, reflecting the membrane potential at the mesoscopic scale, propagates at time 249.8–262 ms and again 317.9–340 ms from SSY to the border between areas 17 and 18 (from Roland, 2010; by permission).

Neurons sending action potentials to another cortical area increase the d*V*_m_(*t*)/d*t* of the target neurons, no matter what caused the action potentials in the first place (Roland, 2010). For example, in the sleeping and anesthetized brain, an up-state in one area may spread to other areas (Figure 1). Up-states typically lasts 1 s or more, during which period the *V*_m_(*t*) is around −50 mV and accompanied by an increased number of action potentials (Steriade et al., 1993; Paré et al., 1998; Destexhe et al., 1999; Petersen et al., 2003b). In the anesthetized and the awake brain, many action potentials are not related to external sensory events (Destexhe, 2011). Traditionally, this is called “spontaneous ongoing activity”, as the sources of this activity are not known. This intrinsic activity is also communicated between cortical areas (Arieli et al., 1995; Lippert et al., 2007; Xu et al., 2007).

To get a full understanding on how d*V*_m_(*t*)/d*t* and spiking dynamics evolve among the cortical areas, one must know the sources and the targets. In principle, this may be possible in studies of anesthetized brains, where it is sometimes possible to capture the population of neurons bifurcating into an up-state (Figure 1). Then one can follow how the up-state spreads to populations of neurons in adjacent areas (Lilly, 1954; Lippert et al., 2007; Xu et al., 2007; Huang et al., 2010; Gao et al., 2012; Zheng and Yao, 2012). In contrast to sensory evoked activity, the spontaneous up-states may spread from different origins in the cortex. The spread out from the initiation site is often in the form of wave fronts of net-excitation traveling over the cortex (0.001–0.2 mm ms^−1^), sometimes the waves have spiral character (Huang et al., 2010). The wide range in the velocity of propagation indicates that the mechanisms behind the spread can be monosynaptic at times (Figure 1) and polysynaptic at other times, or combinations of mono- and polysynaptic progressions.

In the awake state, not surprisingly, there may be spontaneous cortico-cortical communications of *r*(*t*)s in sensory cortical areas and in motor areas although the animal remains relaxed, immobile, and does not receive any external sensory stimuli (Ferezou et al., 2006, 2007; Han et al., 2008; Zagha et al., 2013). Surprisingly even in the awake state, d*V*_m_(*t*)/d*t* increases may also move as wave fronts from sensory to motor areas or vice versa, or between visual areas similarly to the spontaneous up-states. Again the velocity of this cortical propagation is highly variable. The direction of propagation in some cases however mimicks that of sensory evoked d*V*_m_(*t*)/d*t* increases or motor associated (whisking) d*V*_m_(*t*)/d*t* increases (Ferezou et al., 2006, 2007). All examples of spontaneous propagating d*V*_m_(*t*)/d*t* increases between cortical areas were captured by simultaneous measurements of changes in the membrane potentials of populations of neurons in the upper layers of cortex with voltage sensitive dyes. The dye signal change has a near linear relationship to the change in membrane voltage, recorded intra-cellularly *in vivo* from cells in superficial cortical layers (Petersen et al., 2003a; Ferezou et al., 2006; Berger et al., 2007). Furthermore, according to a recent estimate, approximately 90–95% of the dye signal reflects changes in synaptic activity (Berger et al., 2007). Given these premises, Eriksson et al. (2008) showed that significant increases in the temporal derivative of the dye signal *in vivo*, d*V*SD(*t*)/d*t*, can be interpreted as net excitation of the stained membranes and significant deceases as net inhibitions. This means that the investigators in these studies most likely observed the spatio-temporal dynamics of net-excitations of membranes in the upper layers of cortex traveling between cortical areas. The net-excitations could be indirect indications that *r*(*t*)s from one area were communicated to the target area(s). However, the sources of these communications are not known, as the dye signal recordings were not paired with simultaneous *r*(*t*) recordings. Even in the case where one directly observes that the neurons bifurcate into an up-state at a particular spot from where the depolarization spreads out, one must have laminar electrodes at the spot to find the source of increased spiking (which of course could be in the spot itself). Finding the spiking source of spontaneous activity that propagates between cortical areas may in practice involve an electrode density that is unrealistic. See also Chicharro and Ledberg (2012) for theoretical limitations of interpreting causal influences in studies of temporal dynamics of cortico-cortical communications.

Faced with the practical problems, the fact that the cortex has a rich and diverse spontaneous and intrinsic activity, and the microscopic likelihood of finding the sources of the *r*(*t*)s, neuroscientists have thought of ways in which the sources of the dynamics can be controlled. There are basically two strategies: computational models, and experimenter-controlled natural sensory perturbations of the cortex network.

## Current state in the computational modeling of neural signal propagation

Tremendous advances in IT hardware have made it possible to model neural networks of a scale approaching that in the real brain. Realistic computational models of the cortical neuron networks have the great advantage that all sources, synapses, and target neurons are known. Consequently the fundamental variables *r*(*t*) and d*V*_m_(*t*)/d*t* can be observed in any neuron and hence a detailed description of the evolving communication dynamics should be possible. With an estimated average convergence and divergence rate of cortical neurons in the order of 10^4^–10^5^ inputs and outputs (Braitenberg and Schüz, 1998), realistic models even of small cortical patches require the inclusion of several 10,000s of neurons (Potjans and Diesmann, 2012). Hardware progress has allowed modeling of such large populations with some degree of realism in the local dynamics, that is, as biophysical or spiking neurons. For example, Izhikevich and Edelman (2008) modeled a population of 10^6^ phenomenological spiking neurons and linked them in a multi-scale fashion by almost half a billion synapses, combining long-range connections estimated from diffusion imaging of the human brain at the large-scale with the “canonical” microcircuit from cat visual cortex (Binzegger et al., 2004) at the local scale. After adjustment by spike-time dependent plasticity, the network showed self-sustained activity in the absence of external inputs, which activity was organized into different dominant frequencies within different regions and layers. Moreover, the model exhibited propagating waves of excitation and simulated fMRI signals showing slow oscillations with multiple anticorrelated modules, similar to real data. More recently, Potjans and Diesmann (2012) presented a full-scale model of the canonical cortical microcircuit, comprising 80,000 spiking neurons and 0.3 billion synapses, which produced spontaneous asynchronous irregular activity and cell-type specific firing rates in agreement with *in vivo* recordings in awake animals. On a larger scale, the Human Brain Project (Markram, 2012)^1^ is now under way and aims to build a model of the whole brain based on biophysical neurons, that is, including channels characteristics and other features at the molecular scale. While the promise of this enormous modeling effort is that multi-faceted dynamic phenomena may be found at multiple scales, a deeper understanding of such phenomena may also be hampered by the model complexity.

Alternatively, if the main goal of a neural network model is to understand the fundamental relationship between network topologic features and propagation of excitation, smaller models and more simplified assumptions about the local nodes may suffice. For instance, it can be shown with multi-scale models as well as simple excitable nodes (akin to cellular automata) that topological features of brain networks strongly shape brain dynamics. For instance, modular and hub features of biological neural networks induce a modular and target wave-like propagation of excitation, respectively (Zhou et al., 2006; Müller-Linow et al., 2008; Lohmann et al., 2010). “Nodes” in these models correspond to neural elements ranging in scope from individual cells to large-scale populations (e.g., cortical areas).

The question of how the topology of structural connections shapes cortical communication dynamics is addressed by several papers of the Special Research Topic “Cortico-cortical communication dynamics” (Roland et al., 2014). The references to these contributions are underlined. For instance, Mišić et al. (2011) demonstrate through the analysis of functional connectivity derived from EEG data, that the variability of signals at different network nodes (as assessed by the measure of multi-scale entropy) depends on the placement of the nodes within the network. In biological neural networks, which have a non-regular and non-random organization (Sporns et al., 2004), not all nodes are created equal. In particular, some nodes possess more connections, turning them into so-called hub nodes, which are also more central in the network topology. From the observations by Mišić et al. (2011), it also turns out that more central hub nodes have higher signal variability. This finding complements previous experimental and modeling observations that hub nodes also have higher activity than other nodes, which in turns makes them more liable to injury (Buckner et al., 2009). Based on the analysis of MEG data in a visual, face recognition task, Vakorin et al. (2011) showed that the amount of information transferred from one node (i.e., a MEG source) to another was correlated with the difference in variability between the dynamics of these two sources. These results and similar outcomes from analyses of synthetic data suggest that both time delay and strength of coupling can contribute to the relations between variability of brain signals and information transfer between sources. Delay times as well as density and type of coupling were also found to be essential factors by Li and Zhou (2011) who used computational modeling, based on integrate and fire neurons or a neural mass model, to explore factors resulting in anti-phase oscillations between two network modules. The modeling also showed that interactions between slow and fast oscillations may provide a basis for anti-phase synchronization of slow oscillations at small delay times. This work deepens the understanding provided by previous computational models attempting to reproduce functional connectivity during spontaneous activity of the brain (e.g., Deco et al., 2009).

In humans, the neuroanatomical network structure is typically inferred from variants of diffusion tensor imaging and tractography techniques (see Jones et al., 2013 for a sober evaluation). The resulting anatomical matrix expresses the likelihood or density with which two different brain areas are connected through white matter fiber tracts. The second component of the models is the type of dynamics that is assumed for the local nodes. Some neurodynamical models considered a simple oscillatory dynamics (Ghosh et al., 2008; Deco et al., 2009; Cabral et al., 2011), others a more realistic spontaneous state dynamics (Honey et al., 2009), and finally, even very detailed and realistic local networks considering excitatory and inhibitory populations of spiking neurons coupled through realistic NMDA, AMPA and GABA synaptic dynamics, have also been formulated (Deco and Jirsa, 2012).

Further, van den Berg et al. (2012) studied the evolution of random networks of interacting nonlinear dynamical systems in which the coupling between the local dynamical nodes follows a rule of adaptive rewiring. For a large enough number of connections, the system evolves towards a small-world network architecture similar to the one observed in healthy brains after development. Nevertheless, if the number of connections is not larger than a critical value, the system evolves towards a random network. They relate this failure with the fragmentation hypothesis underlying schizophrenia. This study is a beautiful example of how computational and theoretical analysis of dynamical systems serves to deepen our understanding on the relationship between function (activity), structure (anatomy) and development (rewiring). Kiebel and Friston (2011) investigated the reorganization and pruning of synaptic connections in a neuropil stimulated by spatiotemporal input sequences. They demonstrated that the reorganization underlies an optimal Bayesian principle, namely the minimization of free-energy. They were able to show that following this reorganization optimal principle, dendrites self-organize and replicate two key experimental findings (Branco et al., 2010) on directional selectivity and velocity-dependent responses. Banerjee et al. (2012), review different measures characterizing functional and effective connectivity, in particular in MEG data. Furthermore, they propose and show how MEG measurements could be validated by combining the empirical data analysis with simulations of large-scale neurobiological realistic modeling.

## Attempts to follow sensory evoked cortico-cortical communication dynamics. Dependence on the state of the target neurons

**Figure 2:**
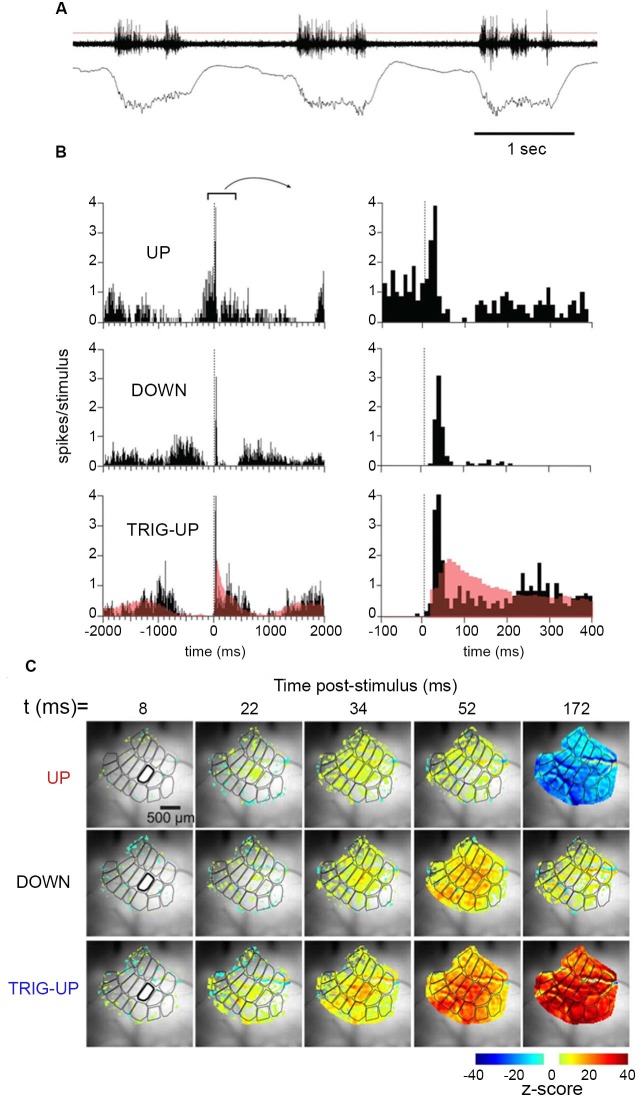
**Temporal dynamics of multiunit activity and local field potentials, and spatio-temporal dynamics of the voltage sensitive dye signal in the barrel field of the mouse during up-state and down-state**. Top: **(A)** Spontaneous multi-unit activity and local field potential at the D 2 barrel during three consecutive up-states. **(B)** Multi-unit activity after stimulating the whisker at 0 ms during an up-state, in the first half of a down-state, and in the last part of the down state. Note the different time scales. **(C)** The spatio-temporal spread of the increase in population membrane potential (voltage sensitive dye signal), after whisker stimulation during an up-state, in the first half of a down-state, and in the last part of the down state (from Civillico and Contreras, 2012). Notably the whisker stimulus only modifies the oscillation in one cycle, but does not alter the future oscillations.

In later years scientists have become increasingly aware that the spontaneous and intrinsic ongoing fluctuations in the membrane potentials and firing of action potentials have a profound effect on sensory evoked activity when it arrives to primary sensory areas (Destexhe, 2011). For example, it has been debated whether sensory evoked *r*(*t*) and d*V*_m_(*t*)/d*t* increases are favored by up-states or down states (Steriade et al., 1993; Contreras et al., 1996; Paré et al., 1998; Destexhe et al., 1999; Petersen et al., 2003b; Crochet and Petersen, 2006; Haider et al., 2006; Luczak et al., 2007). Up-states are associated with high inhibitory and excitatory conductances; whereas in down-states the conductances are smaller, but often coupled to a leak conductance (Contreras et al., 1996; Haider et al., 2006). Civillico and Contreras (2012) induced oscillation between a down-state and an up-state with ketamine-xylazine. They then examined how the phases of the up-state and down-state affected the arrivals of *r*(*t*)s from thalamus and the membrane potentials in the barrel cortex. They found that the local field potentials, the membrane potential changes and the multi-unit activity in the barrel cortex increased less to a whisker stimulus applied during the up-state, as compared to whisker stimulus applied in the later part of the down-state (Figure 2). When the whisker stimulus was given when the membrane was maximally hyperpolarized or when the hyperpolarization diminished in the oscillatory cycle, the whisker stimulus almost invariably triggered an up-state during which the amplitude of the local field potential, the membrane potential and the multi-unit activity was strong (Figure 2). Also the spreading of the depolarization to the whole barrel field was much stronger.

**Figure 3:**
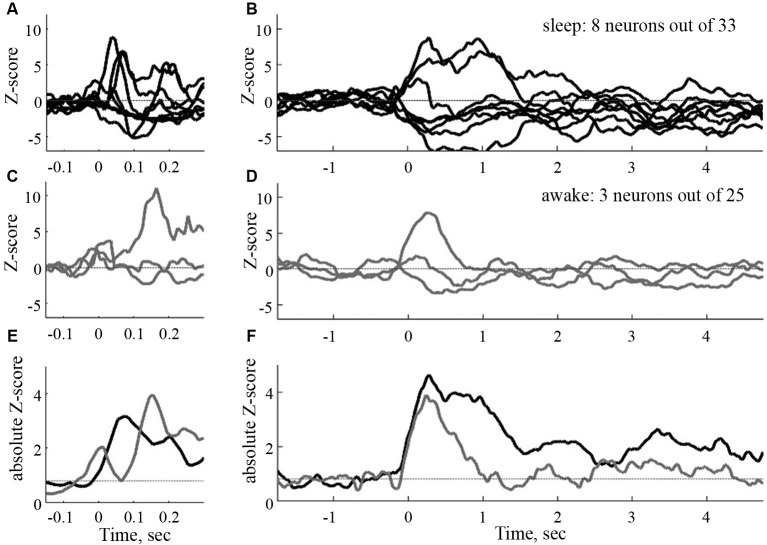
**Time courses of the sound evoked post stimulus histograms in the CA1 of the hippocampus of the rat**. To the left, normalized filtered post stimulus time histograms (PSTHs) at a short time scale (window 50 ms). To the right, same responses at a large time scale (window size 500 ms). Black curves: sleep, gray curves: awake. **(E)** and **(F)**: absolute mean rates in the two conditions (from Vinnik et al., 2012).

Many cortical areas send (multi-synaptic) communications via the entorhinal cortex to the hippocampus (van Hoesen et al., 1972). In awake animals, novel sounds evoke 50 ms latency, short lasting spike trains in hippocampus (Christian and Deadwyler, 1986). Overlearned sounds, if task relevant, may also modulate spiking in hippocampus, albeit often with long latencies 150–300 ms (Itskov et al., 2012; Vinnik et al., 2012), However if the sounds irrelevant for a task, they modulate the spiking in only a few percent of hippocampal neurons also with long 150–300 ms latencies in the awake animal (Vinnik et al., 2012; Figure 3). Surprisingly, if the animals are asleep, 25% of the hippocampal neurons react with short 50–70 ms latencies and long lasting *r*(*t*) increases or decreases even to task irrelevant sounds (Figure 3). These results show that the access to hippocampal neurons is state and context dependent. The sounds did not arouse the EEG, suggesting that the effect, at least partly, may be cortico-cortical, although it is not clear whether the sleep stage had any influence on the accessibility.

**Figure 4:**
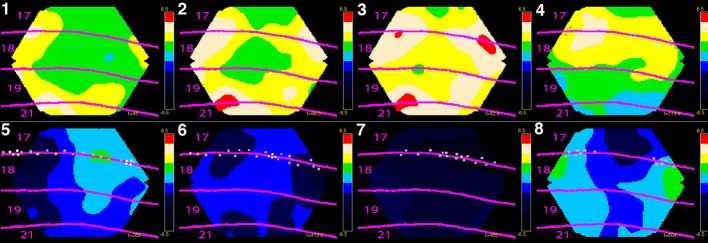
**Eight phases of dynamics of net-excitation, net-inhibition (obtained with voltage sensitive dyes) and multi-unit activity in the cerebral cortex of the ferret exposed to two bars in the field of view moving towards each other**. Mean temporal derivative of the population membrane potential (related to d*V*_m_(*t*)/d*t*) in cytoarchitecturally defined cortical areas 17, 18 , 19 and 21 of the ferret. Mean of three animals shown. **(1)** The two bars have not yet entered the part of cortex monitored by the photodiode camera, but the net-excitation especially in areas 19 and 21 has. **(2)** The mapping of the bars in areas 19/21 has entered the part of cortex monitored. The net-excitation *ahead* of the spiking neurons at the area 17/18 border from the two sides meet at the cortical zone mapping the center of field of view. **(3)** Feedback from areas 19/21 to areas 18 and 17 begin. **(4)** The bars are now separated by 15^˚^ in the field of view and the neurons start to spike at the edge of in the cortex monitored (gray dots). **(5)** The bars are separated by 7.5^˚^ and the neurons at zone mapping the center of field of view start to fire. The positions with more than 90% of the maximal firing rate (the mapping sites) are marked with white dots. **(6)** There is now only one cortical mapping site at the 17/18 border, corresponding to the fact that the bars now occlude one another. Net-inhibition now dominates the cortex at the former mapping sites. **(7)** The net-inhibition is maximal 70 ms after the occlusion in the field of view. **(8)** The net excitation recovers somewhat at the sites of the mapping (bars now drifting apart by 9^˚^), but the spiking remains reduced. The color scale ranges from −6.5 10^−6^ to 6.5 10^−6^ (for laminar propagation see Harvey and Roland, 2013).

Finally, Harvey and Roland (2013), explore experimentally, by using voltage-sensitive dyes, the propagation of activations in the ferret visual system in response to colliding visual stimuli, and how the propagation may be shaped by cortical connections, in particular their direction from primary visual cortices to higher-order cortical areas or in the opposite direction (Figure 4). Anatomical projections proceeding in these two directions have well known orderly characteristics of laminar projection origin and termination (Felleman and van Essen, 1991), but it still remains a challenge to understand the impact of these anatomical features on cortical communication dynamics (Bastos et al., 2012).

## Evolution of sensory evoked cortico-cortical communication dynamics

In a classical approach to follow the cortico-cortical communication dynamics scientists stimulated the sensory apparatus with a very brief stimulus and recorded action potentials or multi-unit activity with laminar electrodes in one or more cortical areas. Typically such an effort result in an ON response, a fast increase in the number of action potentials over some 20 ms, in the primary sensory area. If the stimulus is sufficiently strong, ON responses will spread to many (higher order) sensory areas. In general, however, these studies failed to reveal any clear order of the start of the ON *r*(*t*)s in most cortical areas. For example in the visual areas there were no significant latency differences between the primary visual area neurons in layer 4 and the neurons in areas MT/V5, middle superior temporal visual area (MST) and the frontal eye fields (Best et al., 1986; Schmolesky et al., 1998; Schroeder et al., 1998; Bullier, 2001; Chen et al., 2007). One exception are the progression of ON *r*(*t*)s in V1,V2,V4 and inferior temporal cortex, where the mean ON *r*(*t*)s are separated by approximately 10 ms (Nowak and Bullier, 1997; Schmolesky et al., 1998; Schroeder et al., 1998; Chen et al., 2007). However, the number of potential sources altering the cortical dynamics are many already at the time, approximately 45 ms after the stimulus onset, when the majority of the ON *r*(*t*)s leave the primary visual cortex.

There have been a number of interesting studies in which paired electrode recordings were made in two or more areas that were known to connect anatomically, for example visual areas V1 and V2, V1 and MT/V5, and auditory areas A1, A2 (Movshon and Newsome, 1996; Nowak et al., 1999; Eggermont, 2000; Valentine and Eggermont, 2001). These studies give some insight in the development of temporal dynamics between the two areas, and may reveal likely sources (Movshon and Newsome, 1996). However, the spatial dynamics, and the simultaneous temporal dynamics of the neurons in all the other cortical areas cannot be revealed by this method.

After staining the cerebral cortex with voltage sensitive dyes one can in practice observe some spatial evolution of cortical dynamics of the *V*_m_(*t*) and d*V*_m_(*t*)/d*t*, at least in the upper layers of cortex. This seemingly contradicts the results of the action potential studies just described. Part of the explanation might be that the dye signal *in vivo* reflects synaptic activity at the mesoscopic scale, whereas the action potential recordings capture the activity of single neurons (Lippert et al., 2007; Eriksson et al., 2008). Nevertheless, in several studies one can follow how net increases in the synaptic activity propagate over the cortical areas when the cortex is perturbed by a sensory transient (Senseman, 1996; Prechtl et al., 1997; Senseman and Robbins, 2002; Slovin et al., 2002; Grinvald and Hildseheim, 2004; Roland et al., 2006; Ferezou et al., 2007; Lippert et al., 2007; Xu et al., 2007; Ahmed et al., 2008; Han et al., 2008; Takagaki et al., 2008; Yoshida et al., 2008; Harvey et al., 2009; Ayzenshtat et al., 2010; Meirovithz et al., 2010; Ng et al., 2010; Polack and Contreras, 2012; Harvey and Roland, 2013). This synaptic dynamics may show some order in the feed-forward propagation of net-excitation for example between V1 and V2 in monkeys, rats and turtles, between the barrel field and the motor cortex in the mouse, and between visual areas 17, 18 and 19, 21 in the ferret. Typically the higher order area(s) lag the primary areas with some 8–15 ms depending on species.

Some of these studies contain observations of a reverse order of synaptic propagation, that is, from higher areas towards the primary sensory areas, some 40–50 ms later, i.e., 80–100 ms after the stimulus onset (Roland et al., 2006; Lippert et al., 2007; Xu et al., 2007; Ahmed et al., 2008; Takagaki et al., 2008; Yoshida et al., 2008; Harvey et al., 2009; Ayzenshtat et al., 2010; Ng et al., 2010; Lim et al., 2012; see also Zheng and Yao, 2012; Harvey and Roland, 2013). This mode of propagation has been named feedback. The sources of these feedbacks are not known (but see Zagha et al., 2013). As the synaptic net excitation during feedback propagates fast (0.15–0.25 mm ms^−1^) over the cortex, it was suggested that feedback axons from higher order areas made synaptic contact during their way back from the higher order area. This propagation velocity, though, is slower than that of 1–3 mm ms^−1^ measured in primate axons running from V2 to V1 (Girad et al., 2001), suggesting that higher areas may influence lower areas with different mechanisms.

One major finding from the voltage dye studies was that the dynamics of the d*V*_m_(*t*)/d*t* evolved to engage whole sensory cortical areas within 100 ms after the sensory stimulus. In the barrel field of mice and rats this happened 16–36 ms after the start of stimulation of single whiskers (Derdikman et al., 2003; Petersen et al., 2003a; Civillico and Contreras, 2006, 2012; Ferezou et al., 2006, 2007; Lippert et al., 2007). The whole primary auditory cortex was engaged in 26–40 ms after stimulus start in guinea pigs (Horikawa et al., 1998; Kubota et al., 2012). The whole craniotomy exposed part of the primary visual cortex in ferrets, cats, and monkeys became engaged 48–70 ms after stimulus start, even with small stimuli (Slovin et al., 2002; Jancke et al., 2004; Eriksson and Roland, 2006; Roland et al., 2006; Sharon et al., 2007; Eriksson et al., 2008; Harvey et al., 2009; Ayzenshtat et al., 2010; Meirovithz et al., 2010; Roland, 2010; Chavane et al., 2011; Reynaud et al., 2012; Harvey and Roland, 2013). In mice and rats it took some 70–110 ms for the dynamics to engage the whole primary visual cortex (Xu et al., 2007; Han et al., 2008; Gao et al., 2012; but Lim et al., 2012: 46 ms; Polack and Contreras, 2012). The engagement of the whole area lasted some 60–70 ms, i.e., up to 140 ms after the start of the stimulus, even after very short stimuli (Eriksson et al., 2008). This is the relevant time scale for perceiving changes in the sensory environment (Thorpe et al., 1996).

## Concluding remarks

To measure the evolution of cortico-cortical communications, first one must identify the neurons that communicate their action potentials between cortical areas. Then one must measure how these neurons spread their action potentials to neurons in other cortical areas under natural conditions. Finally one must measure the effect of this communication in the target neurons, i.e., measure the d*V*_m_(*t*)/d*t*, because the temporal evolution of the d*V*_m_(*t*)/d*t* affects the future dynamics of the target neurons. The experience, from experiments and large-scale models of the brain (cerebral cortex), is that the measurements should be done in different scales, from the single neuron scale to the mesoscopic scale (larger populations of neurons), because spiking from a few neurons can spread through cortical layers and evoke spiking in many cortical areas. Moreover, sensory evoked spiking in cortical areas tends to increase d*V*_m_(*t*)/d*t* in a large part or a whole cortical area. This means that the task is to measure the *spatio*-temporal dynamics, at least of the fundamental variables *r*(*t*) and d*V*_m_(*t*)/d*t* from the single neuron to the large population of neurons scale during natural conditions. As discussed, neuroscience so far does not have efficient methods to do this.

In the case of sensory evoked *r*(*t*) one has a chance to identify the neurons in the primary mammalian sensory area starting to send their action potentials to other areas. But what about the neurons starting a thought or starting planning an action? To get insight into this type of cortico-cortical communication dynamics one must monitor neurons in all layers and all cortical areas with sufficient spatial density. The available experimental results show that already 20–30 ms after the start of sensory evoked spiking in cortex 10000’s of neurons may be spiking and perhaps two orders of neurons in addition will have changed their membrane potentials. Furthermore, a fair proportion of these spiking neurons will mutually affect each other across area borders. At this point of time, causal relations of spiking, i.e., which neuron drives which neurons, are not so clear. This problem of understanding the cortico-cortical communication dynamics at the single neuron scale while the communications evolve, experimental neuroscience shares with large-scale computational models of the cerebral cortex and models of whole brains. One, speculative, solution of this conundrum would be if the collective dynamics of the *r*(*t*) and d*V*_m_(*t*)/d*t* of larger populations after the initial evolution reduced the importance single neuron *r*(*t*) dynamics. Thus by observing larger scale spatio-temporal dynamics of these variables one might hope to observe spatio-temporal patterns giving hints of what the brain will perceive or do (Roland, 2010). Such speculations notwithstanding, advances in experimental methods are prerequisites for understanding cortico-cortical communication dynamics.

Science is not there yet.

## Conflict of interest statement

The authors declare that the research was conducted in the absence of any commercial or financial relationships that could be construed as a potential conflict of interest.

## Footnotes

^1^www.humanbrainproject.eu
